# Methodological evaluation of human research on asthmagenicity and occupational cleaning: a case study of quaternary ammonium compounds (“quats”)

**DOI:** 10.1186/s13223-019-0384-8

**Published:** 2019-11-21

**Authors:** Judy S. LaKind, Michael Goodman

**Affiliations:** 1 LaKind Associates, LLC, 106 Oakdale Avenue, Catonsville, MD 21228 USA; 2 University of Maryland School of Medicine, Baltimore, MD USA; 3 Rollins School of Public Health, Emory University, 1518 Clifton Rd, Atlanta, GA 30322 USA

**Keywords:** Quats, Quaternary ammonium compounds, Asthma, Disinfecting, Cleaning, Occupational

## Abstract

In this paper, we review methodological approaches used in studies that evaluated the association between occupational exposure to quaternary ammonium compounds (quats) and occupational asthma. This association is of interest because quats are a common active ingredient of disinfectants and have been linked to work-related asthma in some circumstances. However, any evidence-based assessment of an exposure-outcome association needs to consider both strengths and limitations of the literature. We focus on publications cited by various US and international organizations. Eighteen investigations included in the review fall into two broad categories: case reports and challenge studies of individual patients and population studies that examined the association between quats and asthma occurrence in groups of subjects. We evaluated these studies guided by questions that address whether: exposure data on specific quat(s) and other agents that may cause asthma were included, new asthma cases were differentiated from asthma exacerbation, and information on respiratory sensitivity versus irritation was given. We also assessed consistency across studies. Studies of individual patients, particularly those that provided detailed information on challenge test results, document cases of asthma induced by exposure to quats. By contrast, studies of occupational groups with the highest potential for quats exposure (e.g., cleaners and farmers) do not consistently report increased incidence of asthma due specifically to quats. The unresolved methodological issues include: poor understanding of exposure pathways considering that quats are non-volatile, lack of quantitative data allowing for identification of an asthmagenicity threshold, insufficient information on whether quats are sensitizers or act via dose-dependent irritation or some other mechanism, and inability to quantify risk of new-onset asthma attributable to quats. Another important area of uncertainty is the lack of information on the specific quats being used. There is also a lack of data capable of distinguishing the effects of quats from those of other chemical and biological workplace exposures. The current state-of-the-science does not allow a proper assessment of the potential link between quats and occupational asthma.

## Background

Asthma is one of the most common chronic diseases in adults of working age [1], with about 15% of adult-onset cases attributed to workplace exposures [2, 3]. Work-related asthma includes two different categories: work-exacerbated asthma and occupational asthma [4, 5]. Work-exacerbated asthma is a pre-existing condition that worsens due to the work environment [6]. In contrast, occupational asthma is defined as “asthma due to conditions attributable to work exposures and not to causes outside the workplace” [7].

An increased risk of occupational asthma is well documented in some groups of workers such as professional cleaners [8]. As cleaners can be exposed to a wide array of chemicals in cleaning and disinfecting products as well as dust, mites and various microorganisms [9], effective control of asthma in this population requires a solid understanding of comparative risks attributable to these exposures. One such exposure is that associated with disinfectant chemicals; here we focus on quaternary ammonium compounds (quats).

Quats are the most common active ingredient of disinfectants found in a variety of settings, especially in health care facilities [10] where they are used for cleaning floors, furniture, and walls [11] as well as for disinfecting medical equipment such as blood pressure cuffs [11], surgical instruments and endoscopes [12]. Quats chemistry has evolved over the years with the goal of improving activity against microorganisms [12]. Two main categories of quats are alkyl dimethyl benzyl ammonium chloride (ADBAC) [13] and didecyl dimethyl ammonium chloride (DDAC) [14].

Various agencies have described quats as known asthmagens, and have made recommendations regarding reductions in their use [15–17] (although these agencies do not provide support for their recommendations, several national and international organizations have reviewed the literature on this issue; see “Approach to the methodological evaluation” section). At the same time, quats play an important role in disinfection and for this reason a better understanding of the data that prompted their designation as “known asthmagen” is warranted. In this paper, we examine methodological approaches used in studies that are often cited as the basis for this designation.

These studies deserve a critical review because of several issues that need to be considered in making evidence-based decisions about quats and asthma in the workplace. First, quats are not a single chemical but rather a large group of chemicals. Various formulations may contain several forms of quats and their composition has evolved over time. Second, as components of cleaners and disinfectants [12], quats are usually encountered together with other potentially sensitizing or irritating chemicals [18–20] as well as various agents not associated with the cleaning/disinfecting products themselves. Third, it is not entirely clear if quats are capable of causing asthma in a previously healthy individual or whether they trigger asthma exacerbation. While work-exacerbated asthma may be associated with a wide range of non-specific factors including extreme temperatures, humidity, physical exertion or emotional stress [21, 22], occupational asthma is caused in a previously healthy individual via exposure to a specific workplace agent [7, 23–25]. Fourth, quats have been described in the published literature as sensitizers [5, 8, 26–30] or as irritants with either immediate or delayed response [5, 31]. Although sensitizer- and irritant-induced asthma may not be mutually exclusive and may be difficult to distinguish clinically [32], from industrial hygiene, exposure control and prevention perspectives, the distinction may be important because it can indicate different response thresholds with a sensitization threshold being so low that it may require removal from any exposure. Fifth, as occupational use of quats is widespread, estimating the likelihood of quats-induced asthma is necessary to quantify the potential public health burden and to inform the need for and extent of exposure control measures. These five issues and the proposed ways of addressing them are discussed in this article.

## Approach to the methodological evaluation

It is important to note that this evaluation was not designed as an exhaustive review of all available data, but rather as an examination of strengths and weaknesses of human studies cited as basis for conclusions in key agency and professional society reports. We focus on the following reviews of the literature on asthma and quats by the following organizations: US Environmental Protection Agency (USEPA), the Centers for Disease Control and Prevention (CDC), National Institutes of Safety and Health (NIOSH), the European Academy of Allergy and Clinical Immunology and the American Thoracic Society:

- Two US EPA reports [13, 14].
- A CDC Guideline document [11].
- NIOSH National Occupational Research Agenda Cleaning and Disinfecting in Health Care (CDHC) Working Group [33].
- A consensus statement by a European Academy of Allergy and Clinical Immunology task force [8].
- An Official American Thoracic Society Workshop Report [5].

The literature referenced in these documents included 14 publications. In its Final Work Plan for didecyl dimethyl ammonium chloride (DDAC) and alkyl dimethyl benzyl ammonium chloride (ADBAC), the US EPA described various publications that reported on work-related asthma and associations with exposure to quaternary ammonium compounds [34–40]. The CDC relied on a review by Purohit et al. [39] to link occupational asthma with exposure to benzalkonium chloride. The National Institutes of Safety and Health (NIOSH) National Occupational Research Agenda Cleaning and Disinfecting in Health Care (CDHC) Working Group [33] reported that chemicals present in cleaning and disinfecting products—including quaternary ammonium compounds—can cause or exacerbate asthma and reference many of the same publications as noted in the US EPA reports [34, 39, 41–44]. The European Academy of Allergy and Clinical Immunology Task Force concluded that disinfectants including quaternary ammonium compounds are “specific causes of or exacerbation for asthma” [8]. The Task Force cited three publications to support this conclusion [34, 39, 45]. The American Thoracic Society Workshop Report [5] describes benzalkonium chloride as both a specific sensitizer and an irritant and cites two studies [46, 47].

We retrieved these 14 publications and examined their bibliographies; we identified nine additional relevant articles for review [33, 48–55]. Of the total 23 papers, one publication was not considered further because it was not in English [37]; and two other papers [43, 46] were excluded because they were non-occupational studies of nebulizer exposure. Although not directly related to asthma, two papers were retained for further review because they provided useful information on quats exposure [41, 42].

The included publications described 19 separate investigations that fall into two broad categories: case reports/case series and challenge studies of individual patients [33–35, 39, 48, 51, 53] and population studies [36, 38, 40, 44, 45, 47, 49–52, 54, 55] that examined the association between quats and asthma in groups of subjects. A summary of the reviewed studies is given in Table 1.

**Table 1:** Summary of reviewed publications on quats and asthma

References	Study type	Study location	Quats exposure
Burge and Richardson [35]	Single case report/SIC study	UK	Lauryl dimethyl BAC
Bernstein et al. [34]	Single case report/SIC study	USA	BAC
Purohit et al. [39]	Three case reports/SIC studies	France	BAC
Rosenman et al. [51]	Four case reports	USA	ADBAC, DDAC, quaternary ammonium salts
Quinn et al. [33]	Four case reports	USA	ADBAC, DDAC
Vandenplas et al. [53]	Case series [44 subjects]/SIC studies	Belgium	QAC
Bellier et al. [48]	Case series [22 subjects]/SIC studies	France	DDAC, ADBAC, didecylmethyl ammonium propionate, BAC, bis-aminopropyl-laurylamine, amine oxide
Rosenman et al. [51]	Analysis of the SENSOR database	USA	Quaternary ammonia
Mehler et al.	Analysis of the SENSOR database	USA	QAC
Paris et al. [50]	Analysis of the National Occupational Health Surveillance Network	France	QAC
Massin et al. [45]	Cross-sectional study of food industry workers	France	QAC
Gonzalez et al. [36]	Cross-sectional study of health care workers	France	QAC
Dumas et al. [49]	Cross-sectional study of hospital workers	France	QAC
Weber et al. [40]	Retrospective cohort study of persons employed in a large health care system	USA	Not specified
Preller et al. [62]	Cross-sectional study of pig farmers	Netherlands	QAC
Vogelzang et al. [44, 54, 55]	Cross-sectional studies of pig farmers	Netherlands	QAC
Smit et al. [52]	Cross-sectional studies of conventional and organic farmers	Netherlands	QAC

*ADBAC* alkyl dimethyl benzyl ammonium chloride, *BAC* benzalkonium chloride, *DDAC* didecyl dimethyl ammonium chloride, *QAC* quaternary ammonium compound

Each category of studies listed in Table 1 is evaluated separately, guided by the following broad questions:

1. Does the study provide exposure data on the specific quat(s)?
2. Is the study design able to distinguish between exposure to quats and other agents that may cause asthma?
3. Is there a distinction between new incident asthma and exacerbation of existing condition?
4. Is there evidence that the mechanism of asthma is mediated via respiratory sensitivity or irritation?

Taken together, the above four questions evaluate whether or not quats are capable of inducing asthma in humans. The population-based studies are further evaluated by asking one additional question:

5. Is there evidence of a consistent association between quats and occupational asthma risk and if so, how much (or what proportion) of this risk is attributable to quats in the workplace?

This additional question addresses the broader public health issue of whether or not quats exert a quantifiable asthma burden in occupationally exposed populations.

## Critical evaluation of reports on quats and asthma in individuals

In this section we focus on case studies, case-series and challenge tests. The medical literature generally agrees that a link between a suspected occupational asthmagen and workplace asthma may be confirmed by a positive specific inhalation challenge (SIC) test [5, 56]. If conducted properly with blinding and use of a control agent, an SIC test is similar to a crossover trial. A substance is designated as an “asthmagen” if there is evidence of recurrent bronchospasm following repeated SIC administration and no reaction in response to exposure to a control substance [57].

### Summary of case reports, case/series and challenge studies

The current literature describes several relevant cases of occupational asthma in both the US and Western Europe. These case reports were published between 1994 and 2016 and describe occupational asthma in a variety of settings. The most useful are reports that incorporate SIC test results.

In one of the earliest publications, Burge and Richardson (1994) described a case of occupational asthma attributable to quats exposure in the UK [35]. The patient was a 44-year-old hospital pharmacist who reported his first asthma attack 22 years earlier following a spill of chloroxylenol. He also reported evidence of asthma in response to trichlorophenol exposure. Later he reported worsening of symptoms after starting a new job at a pharmacy that was equipped with a cleanroom manufacturing area. His history revealed most pronounced symptoms after the floor of the pharmacy was cleaned with a product (Vantropol) containing lauryl dimethyl benzyl ammonium chloride as well as several other ingredients. SIC tests revealed a delayed reaction 7 h following exposure to lauryl dimethyl benzyl ammonium chloride (1 mL of solution of agent in 1:400 dilution in gauze), but not to other components of the cleaning agent in question. The report indicates that the symptoms resolved after exposure ended, but “minor” work-related symptoms resumed several months later.

Another early case of quats-related occupational asthma was reported in a 22-year-old US resident who worked in a factory that manufactured house-cleaning products [34]. The patient developed symptoms of asthma after approximately 7 months of employment. Following initial evaluation she underwent single-blind placebo-controlled open-room SIC testing for each cleaning product with potential for workplace exposure (exposure details not provided). Within 5 min after exposure to toilet bowl cleaner with benzalkonium chloride (BAC), the case experienced asthmatic and other symptoms. The quats-specific IgE levels were not elevated.

Three cases of occupational asthma among nurses were reported in France [39]. All three reported symptoms of asthma following exposure to BAC. The reactions occurred in different patients in a variety of exposure scenarios: after preparing a disinfectant containing 10% BAC, cleaning surgical instruments with a disinfectant containing BAC, being exposed to a BAC-containing surface cleaning detergent, entering an area that had been cleaned with a BAC-containing agent, or on entering a room with a solution containing 40% BAC. In one case, the authors specifically pointed out no past history of asthma, in another case there was history of pre-existing upper respiratory allergy and contact dermatitis, and in the third case no information on past history was provided. All three cases tested negative for quats-specific IgE. The three patients underwent closed-chamber SIC testing with BAC-containing detergents in a tray. Neither concentrations of BAC nor the identity of other chemicals in the detergents was given for two of the cases; for the third case, the solution was a 0.1% dilution in water. The quat in the detergent was described variously as BAC or QAC and it is unclear which specific chemicals were used for testing. All three patients had delayed bronchospasm response 3–14 h following exposure.

In a US-based case series, Rosenman et al. described four patients who were diagnosed with occupational asthma linked to workplace exposure to various cleaning agents [51]. The cases were identified through the Sentinel Event Notification System for Occupational Risks (SENSOR) database, which collects data on occupational asthma in the states of California, Massachusetts, Michigan and New Jersey. Two of the four cases reported exposure to quats; both were employed as hospital housekeepers. One case reported reaction following exposure to three different products, one of which contained two quats (*n*-alkyl dimethyl benzyl ammonium chloride and didecyl dimethyl ammonium chloride) and the other case to a floor cleaner containing quaternary ammonium salts, ethyl alcohol, and sodium hydroxide. None of the cases underwent a SIC test, and there was no evidence that IgE testing was performed.

Most recently, a review article reported four occupational asthma cases linked to quats exposure [33]. While these case reports were described in some detail, information on diagnostic testing or—for two of the cases—on type of quats products used was not provided. Two cases of “work-related asthma” were reported at a hospital in Massachusetts. Following an investigation, both cases were attributed to cleaning and disinfecting with quats and both had to stop work in the operating room where the exposure occurred. No additional information about these two cases is available. The third case was a hospital environmental services worker in Michigan who reported developing asthma immediately after the introduction of a cleaning product containing didecyl dimethyl ammonium chloride and alkyl dimethyl benzyl ammonium chloride. The fourth case was a medical records clerk in California who reported difficulty breathing when coming in contact with surfaces cleaned with disinfectant wipes containing alkyl dimethyl benzyl ammonium chloride and dimethyl ethyl benzyl ammonium chloride. Despite stopping work and cessation of exposure, the symptoms persisted and the patient continued to report respiratory difficulty in response to a wide variety of products.

In a larger case series of 44 patients who underwent SIC testing at a specialized clinical center in Belgium, several individuals reported exposure to quats alone or in combination with a different agent (e.g., glutaraldehyde or ethanolamines). SIC tests involved specific cleaning materials and the type of exposure used during test administration was based on participant interviews, Material Safety Data Sheets and information from the Belgian Workers’ Compensation Board. The products were diluted in either cold or hot water, brushed on cardboard, and/or sprayed. SIC test results for quats were positive in 11 of the cases, 10 in response to quats alone [53].

The most recent case series publication presented a chart review of 22 patients who reported asthma symptoms in response to quats exposure [48]. All subjects in the case series underwent an SIC test. The test started with a control challenge using saline solution. The next day, the participants were tested with “pure” quat suspected of causing asthma. The challenge agent was presented as a solution with concentrations ranging from 0.1 to 1%. Exposure occurred after a bucket containing 3 L of diluted product was placed in a chamber. The duration of exposure was increased by 10-min intervals. The challenge test continued until the patient had a 15% fall in FEV_1_ or until exposure lasted a total of 1 h. Approximately half of the patients (12 of 22) were described as having a “positive outcome”; in nine of those, the quat in question was didecyl dimethyl ammonium chloride and for three of the participants the quat was benzalkonium chloride, and in three cases the challenge agent was alkyl dimethyl benzyl ammonium chloride.

### Evaluation of case reports, case/series and challenge studies

In general, case reports are of limited value when assessing a causal hypothesis because perceived temporal proximity of exposure and outcome may be coincidental (Gordis 2004). However, when the disease of interest is asthma, a well-conducted challenge study with repeated administration of an agent and the use of a control substance may demonstrate a causal link between exposure and asthmatic response in an individual even if the data are limited to one person. Taken together, these case reports, particularly those that provided detailed information on SIC testing results, indicate that certain quats can act as asthmagens. Nevertheless, due to the lack of data on levels of exposure or consistent information on specific quats tested, these studies cannot be used to determine whether quats are sensitizers or act via a dose-dependent irritation mechanism [58]. Further, because of a lack of data on exposure levels, it is not possible to discern a threshold below which quats exposure would not be expected to result in an adverse respiratory outcome. The retrospective nature of these case reports (excluding the challenge components) means that these studies are unable to distinguish between exposure to quats and other agents that may be asthmagens. Regarding complete exposure pathways, quats are not volatile and no information was provided on how quats were applied by the cases (wipes, sprays, etc.). Therefore, it is not known whether a complete exposure pathway was present. Moreover, because of inconsistent data on previous history of asthma and allergies, it is not possible to distinguish between true occupational asthma and work-exacerbated asthma. Finally, the description of the cases do not include sufficient details to allow inferences [59].

## Critical evaluation of population studies on quats-asthma associations

In the previous section, we evaluated literature focused on individual cases of asthma in persons exposed to quats. While the best-designed and implemented studies of this type may be capable of showing that a particular agent can trigger an asthma attack in a specific individual, it may not answer important questions about the overall impact of the agent on the workforce. These types of questions can only be addressed in a population-based study. Here we examine the literature on two kinds of population-based studies related to quats exposure: surveillance studies and epidemiology studies.

### Summary of surveillance studies

Surveillance programs are designed to continuously and systematically collect health-related data with specific focus on a particular type or category of disease [60]. This information generally comes from physicians or other health care providers. Surveillance studies of work-related asthma usually include information on the putative exposure factors in each reported case.

In a previously cited publication assessing data from the SENSOR database, Rosenman et al. summarized exposure circumstances of 236 work-related asthma cases in four US states over the period 1993–1997 that were associated with the use of cleaning products [51]. Of those, 188 individuals (80%) were characterized as experiencing new-onset asthma. Among the 188 new-onset cases, 42 had reactive airways dysfunction syndrome (RADS), which typically occurs following a sudden high-level exposure (e.g., accidental spill) and the remaining 146 were characterized as non-RADS occupational asthma. In only 21 cases of new-onset occupational asthma was there was a “known” asthma inducer. A list of reported work-related asthma inducers included unspecified cleaning materials (107 cases), bleach (43 cases), acids, bases and oxidizers (23 cases) and unspecified disinfectants (20 cases). Quats (described as “quaternary ammonia”) were identified as asthma inducers for three cases, although the cases were described more generally as having work-related asthma and it is not known if these were actually cases of occupational asthma. Regarding exposure, no information was obtained on concentrations of quats or how the products were used (e.g., wipe, spray) nor was information provided on whether cases were exposed to mixtures of cleaning and disinfecting agents or other agents (although two of the four cases described by Rosenman et al. indicate that these workers were exposed to various chemicals in addition to quats).

SENSOR database analyses limited to three states (Louisiana, Michigan and Texas) were performed and included information on cases of occupational illness (including ocular symptoms/signs, neurologic, respiratory and dermal) reported in 2002–2007 [38]. Quats were listed as the active ingredients identified in 38% of reported illnesses. The authors also reported that of the 121 cases with respiratory signs or symptoms, 11 were in persons with asthma who experienced an acute attack. In addition, six cases of wheezing were reported in persons without history of asthma. It is not clear how many people with respiratory illnesses had quats exposure, because unlike the earlier analysis of the SENSOR database [51], attribution to various exposures in this study was done for all cases combined.

A more recent French study examined occupational exposures in 2914 work-related asthma cases reported to the National Occupational Health Surveillance Network between 2001 and 2009 [50]. The study evaluated temporal changes in the proportion of cases linked to different workplace exposures. The attribution of cases to a particular exposure was based on a standardized reporting form filled out by a physician. The form included the “main occupational exposure and four other possible agents”. Based on these data, the proportion of cases linked to quats increased from 1.4% in 2001 to 8.3% in 2009; however, it is not clear if this percentage increase was due to increased incidence of quats-associated work-related asthma or decreased contribution from other exposures (e.g., latex). As attribution of cases to a particular exposure is based on physician reporting, an alternative explanation for this observed temporal change is greater awareness about the possible role of quats. Further, the data included all work-related asthma including new onset and asthma exacerbation. No data were available on quats concentrations, product/application type, or assessments of mixtures.

### Evaluation of surveillance studies

Surveillance studies demonstrate that work-related asthma occurs in a variety of settings, although the data may not be able to differentiate occupational asthma from work-exacerbated asthma. The surveillance studies also indicate that a certain proportion of work-related asthma is thought to be quats-related. The most important drawback of surveillance studies is very limited information on exposure (e.g., specific quat compounds used and air concentrations; co-occurring exposures). In addition, a lack of population denominators does not allow for an estimation of the true risk of work-related asthma associated with different exposures. Such information can only be obtained from prospective epidemiologic studies that systematically collect data on asthma incidence in well-defined cohorts of health care workers or other individuals responsible for cleaning and disinfection in various settings.

### Summary of epidemiologic studies

The most valuable population-level information on the relation between workplace exposure and asthma occurrence can be obtained from prospective epidemiologic studies of well-defined cohorts of workers. A less desirable approach is to conduct a cross-sectional study that permits an estimation of prevalence (rather than incidence) of disease. We describe here epidemiologic studies on quats and asthma for three occupations: food industry workers, health care workers and farmers.

A cross-sectional study of food industry workers categorized participants as exposed (n = 175) or not exposed (n = 70) to cleaning and disinfection products [45]. All participants were evaluated with respect to self-reported symptoms of respiratory irritation, results of pulmonary function tests, and evidence of bronchial hyperresponsiveness to metacholine. Exposure to chloramines, aldehydes, and quats was assessed based on air concentrations measured with personal samplers at the time of various tasks. A total exposure index was calculated based on air concentrations and irritant properties of each agent and exposed participants were then assigned to two groups (“exposed” and “more exposed”). All quats air concentrations were below the limit of detection and for this reason quats did not contribute to the total exposure index. The authors observed higher prevalence of various irritation symptoms in exposed workers, especially those in the higher-exposure group. By contrast, there were no associations with pulmonary function tests and methacholine-induced bronchial hyperresponsiveness.

In a French cross-sectional study, 543 individuals employed in various health care settings were administered a questionnaire to collect information on occupational exposure and history of asthma [36]. Exposure to quats and other agents was assessed based on use of cleaning and disinfecting chemicals or products and types of cleaning/disinfection tasks performed. Evidence of sensitization to two agents—quats and latex—was established based on IgE tests. No air concentration data were obtained. The study observed a statistically significant association between exposure to quats and prevalence of physician-diagnosed asthma. The prevalence of self-reported “new onset asthma” was also higher in quats-exposed workers, but the result was very imprecise, not adjusted for co-variates, and was not statistically significant. There was no significant association between positive IgE test for quats and either physician-diagnosed asthma or new-onset asthma. Notably, IgE sensitization to latex as well as self-reports of performing “general disinfection tasks” and “dilution of disinfectants” were significantly related to both self-reported asthma diagnosis and new onset asthma. In addition, asthma diagnosis was also related to general cleaning tasks. Exposure to quats was the sole chemical exposure that remained statistically significantly associated with asthma diagnosis in the multivariable model, although the corresponding adjusted results for new onset asthma are not included. The data presented in this study need to be viewed with caution due to an important methodological limitations: among 700 eligible subjects, only 543 agreed to participate; of those, data were missing on 111 individuals. Thus, the analysis was based on 62% of eligible persons.

In another French cross-sectional study of 1355 hospital workers [49], the authors examined associations between current asthma and occupational exposure to cleaning agents. The authors aimed to address limitations in past exposure assessments by using three methods: a job-specific questionnaire, expert evaluation, and an asthma-specific job-exposure matrix. The data based on self-report were too sparse to allow any analyses because only three cases and nine asthma-free individuals recalled being exposed to quats for at least a day/week. In contrast, expert assessment determined that at least 1 day/week exposure occurred in 31 asthma patients and 65 controls. The resulting analyses demonstrated no association between quats exposure and asthma. When expert assessment further subdivided quats-exposed participants into low or moderate/high intensity categories, neither association was statistically significant, although there was a suggestion that asthma may be more common in the high exposure group.

More recently, Weber et al. performed a retrospective analysis of the occupational clinic records pertaining to persons employed at a large health care system [40]. The outcomes of interest were injuries or illnesses related to chemical exposures and reported over a period of 10 years. The full population under study was estimated using data obtained from the human resources office. The authors reported that over the time period under study the population of health system employees contributed a total of 69,075 work years, although the assumptions and calculations that were used to obtain this estimate are not provided. The authors observed that 70 of 128 chemical exposures incidents were caused by a known germicide; of those, 18 were attributed to quats. The most common specific conditions identified in the records were dermatitis and splashes to mucous membranes whereas “no episodes of acute bronchospasm or persistent asthma were reported related to germicide exposure” (Weber et al. [40]). The methods and conclusions of this study were criticized as inconsistent with national surveillance data [61]. It is worth noting, however, that surveillance data are derived from a much larger population base, and judgements about consistency or inconsistency of results in the Weber et al. article with surveillance studies need to be reserved until the estimates of occupational asthma rates among quats-exposed workers become available.

Farmers are a separate occupational group of interest in terms of quats exposure, especially pig farmers who regularly use disinfectants when cleaning animal housing. Several studies were conducted with pig farmers in The Netherlands. In all of these studies, quats exposure was assessed through the use of questionnaires or other forms of reporting such as telephone interviews. Most of these studies followed the methodology described in Preller et al. [62] by inquiring about the use of disinfectants, and if so, the type of agent utilized for this purpose.

In one of the earliest publications, Preller et al. assessed associations of disinfectant and endotoxin exposure with chronic respiratory symptoms in 194 Dutch pig farmers with and without evidence of atopy [47]. Atopy was defined as evidence of elevated IgE levels specific to one or more common sensitizers (dust mite, grass or birch pollen and cat allergen). Quats were the most commonly used disinfectants, either alone or in combination with aldehydes or chloramine-T. The use of quats was related to asthma-like respiratory symptoms in farmers with atopy, but not in farmers who had no evidence of atopy. Results were adjusted for age and smoking, but not for endotoxin exposure, which typically co-occurs with the use of disinfectants in this setting. No quats-specific measurements were obtained.

In a 1999 publication, Vogelzang and colleagues examined whether there was an association between quats exposure and asthma among pig farmers based on positive responses to the questions “Did you have a wheezing chest for more than one week in the last 2 years?”, or “Did you ever have attacks of chest tightness (asthma)?” In this cross-sectional study, quats and certain aspects of the disinfecting process were associated with self-reported asthma symptoms [55]; however the numbers of cases are not reported and no information was available on levels of quats exposure.

Two other studies by the same group of researchers [44, 54] used histamine challenge to determine if a person exhibited signs of bronchial responsiveness. The outcomes of interest were defined as either “mild bronchial responsiveness” or “clinical hyperresponsiveness” depending on the histamine concentration and the magnitude of change in spirometry measures. In the first analysis [44], there was a statistically significant positive association between use of quats (versus no disinfection) and mild bronchial responsiveness. The authors did not compare prevalence of mild bronchial responsiveness in farmers who used quats and those who used other types of disinfection. No information is available on the association between quats and the second outcome “clinical hyperresponsiveness”, which may be more indicative of asthma.

In the second analysis [54], data on bronchial responsiveness were analyzed longitudinally among 171 pig farmers followed for 3 years. This study addressed the hypothesis that repeated exposure to a possible asthmagen will result in increasing levels of bronchial responsiveness over time. Based on these analyses, the authors concluded that “use of quaternary ammonium compounds as active substance in disinfectants was not associated with increases in responsiveness”, and acknowledged the difference between this finding and the results of their earlier cross-sectional studies.

In a more recent cross-sectional study conducted among Dutch conventional and organic farmers [52], asthma was defined as a positive response to any of the following questions “Have you had an attack of asthma in the last 12 months?”; “Have you been woken by an attack of shortness of breath at any time in the last 12 months?”; and “Are you currently taking any medicine for asthma?”. Compared to individuals who reported no disinfectant use, the association between questionnaire-derived quats exposure and asthma was not statistically significant after controlling for age, smoking habits, family history, and farming-related variables.

### Evaluation of epidemiologic studies

It is important to point out that studies of cleaners and hospital workers cannot be directly compared to those of animal farmers due to differences in exposure modes and patterns. In farming, quats exposure is commonly associated with co-exposure to high levels of organic dust, endotoxin, and microorganisms. Further, unlike cleaning methods that take place in health care settings (e.g., wipes, hand sprays), quats in farming may be delivered in the form of high pressure spray [47].

Perhaps the most important methodological weakness affecting the available studies of asthma outcomes is reliance on recall for exposure assessment. Self-reports can be influenced by the participant’s asthma status or by lack of knowledge of the active ingredients in the products used [63]. A validation study comparing self-report to expert assessment demonstrated a very high likelihood of exposure misclassification, specifically for quats [42]. Using expert assessment as the gold standard, self-report correctly identified only 20% of quats-exposed individuals for both asthma cases and asthma-free participants. Among persons considered not exposed to quats based on expert assignment, exposure was incorrectly self-reported among 20% of asthma cases, but in none of the healthy participants. If confirmed in other settings, these data would indicate that reliance on self-report may provide results that are essentially uninterpretable.

Another methodological weakness of the available literature is the predominance of cross-sectional studies. A key limitation of a cross sectional study is that it ascertains the exposure and disease information simultaneously and cannot measure occurrence of new disease (i.e., incidence) in exposed and non-exposed individuals [64]. Assessing disease incidence requires longitudinal data collection with the ability to identify new cases of asthma in individuals who are confirmed to be disease-free at baseline. To our knowledge, none of the studies of quats-exposed workers used this research design.

On balance, the epidemiological studies do not demonstrate that cleaners or farm workers experience increased incidence of asthma due specifically to quats. Only two of these studies used measurement-based as opposed to self-reported methods to determine quats exposure: one using air concentrations [45] and the other based on IgE tests [36]. Neither demonstrated a link to asthma or related outcomes although it is important to note that Massin et al. [45] did not observe concentrations of quats above the limit of detection.

## Discussion

The identification of workplace asthmagens is a continuous process with multiple new agents identified as possible causes of occupational asthma every year [65, 66]. To our knowledge, one of the most up-to-date lists of occupational asthmagens is maintained by the Committee on Occupational Standards, Equity, Health and Safety in Quebec, Canada [67]. As of February 2019, the list included 527 agents; one of those is BAC. Each entry on the list is accompanied by a reference, usually a case report, and for BAC the single supporting reference is the previously-mentioned report of asthma in a person employed at a house cleaning products manufacturing facility [34].

Our expanded review of the literature identified a total of 37 cases of asthma linked to quats exposure; of those, in 29 cases the diagnosis was confirmed via a SIC test. These cases were reported in a variety of locations across the US and several European countries, and spanned a period of over 20 years. There were only two cases (one from Bernstein et al. [34] and another from Purohit et al. [39]) for which the authors specifically indicate no history of pre-existing asthma. None of the case reports provided data on the levels of quats that triggered the bronchial response.

While quats, or more specifically BAC, have been shown to cause or exacerbate asthma in some individuals, the available case reports are unable to assess the relative importance of these chemicals as occupational asthmagens. Evidence-based judgment about the contribution of quats to the overall burden of asthma requires accurate estimates of absolute and attributable risk of disease in the exposed populations. This type of information can be obtained from prospective population level studies evaluating incidence of asthma among disease-free individuals with documented, well-described and quantified quats exposure.

It is important to acknowledge that persons engaged in cleaning and disinfection activities are reported to experience significantly increased risk of work-related asthma. For example, the European Community Respiratory Health Survey [2], which included 6837 individuals from 13 countries, found that exposure to “cleaning products” was associated with moderately elevated risk of asthma; however, no quats-specific data were available. A more recent study of 21,802 subjects residing in five Northern European countries (Denmark, Iceland, Norway, Estonia and Sweden) also reported elevated risk of asthma among persons that reported working with “cleaning agents” [68], but provide no data on the specific chemicals in this category.

As reviewed elsewhere [19, 69, 70], cleaning materials capable of both inducing and exacerbating asthma include, besides quats, a wide range of irritant materials such as bleach, ammonia and degreasing sprays, as well as other disinfection products such as aldehydes, ethanolamines, glycol ethers and various alcohols. For example, Gerster et al. identified over 132 different chemicals in 105 products as part of a survey of Swiss professional cleaning substances [71]. Exposure to many of these chemicals often occurs at the same time and in the form of complex mixtures [72].

Certain occupational groups may be routinely co-exposed to other asthmagens in addition to cleaning and disinfecting chemicals. For example Malo and Chan-Yeung [31] note that relevant exposures among health care workers may involve not only cleaning and sterilizing agents, but also pharmaceuticals, metals in dental alloys, methacrylates, aerosolized medications and latex. Data also indicate that asthma among persons who perform cleaning and disinfection activities may be triggered by exposures to biological agents such as fungi and endotoxins. For example, in a series of occupational asthma cases diagnosed among professional cleaners in Finland, in most (11 of 20) patients, SIC test was positive for mold, most commonly Aspergillus species. Among the remaining nine cases, asthma was induced by ethanolamines, a cleaning agent containing chloramine-T, isocyanates and nickel sulphate [9].

From the pathophysiology perspective, an asthma-inducing agent is expected to elicit a bronchial response if it is inhaled in sufficient quantity [73]. For this reason, an evaluation of the association between a chemical of interest and asthma risk requires an accurate assessment of inhalation exposure. Several studies described efforts to measure air concentrations of quats with somewhat disappointing results. For the previously-cited study of workers in the food industry [45], sampling was conducted using personal samplers placed on the collars of workers with various sampling times (15 min to 2 h). In addition, area samples were collected. Quats were sampled on tubes containing silica gel, percolated with acetonitrile/potassium dehydrogenate aqueous solution, and analyzed using liquid chromatography [74]. All quats measurements in that study were below the limit of detection (LOD), but the LOD value was not provided.

Vincent et al. [70] used ion chromatography to analyze quats (specifically didecyl dimethyl ammonium chloride [DDAC]) in hospital air during various disinfecting activities, but also reported no measurements above the LOD, which was set at 28 μg/m^3^. The authors concluded that “…the insignificant volatility of DDAC would not seem to be able to contaminate the indoor hospital atmosphere during the disinfection process”.

More recently, LeBouf and colleagues used a mass spectrometry approach to analyzing quat levels in air and surfaces samples. To test their approach, they conducted an experimental field study with a worker actively cleaning a hospital bathroom or sweeping a hospital waiting room [75]. The application of this methodology resulted in detectable levels of 0.15–3.5 μg/m^3^ (depending on the specific quat and the type of activity evaluated); however, it is not clear if the same approach would be feasible for assessing exposure in a population-based study of quats and asthma. According to LeBouf et al. [75], “a major barrier to better understanding exposure–response relationships for quats has been the absence of a robust analytical method that can be used for multiple pathway (surface and air) exposure assessment approaches”.

In addition to the application of quats to surfaces during cleaning and sweeping, inhalation exposure to these chemicals could result from the use of aerosols or from quats sorbed to suspended particles [76]. Another mode of exposure occurs in pig farming, where quats may be delivered in the form of high pressure spray. This presents an important opportunity for research; however, to our knowledge all studies of respiratory endpoints in this occupational group [44, 47, 54, 55] relied on reported rather than measured quats exposures.

A frequently used approach aimed at improving the accuracy of exposure assessment is the development of job-exposure or job-task-exposure matrices (JEM and JTEM, respectively). For example, Quinot et al. [28] reported the development and application of JEM and JTEM in a study of registered nurses. An occupational questionnaire was sent to several thousand female nurses, who provided information on self-reported frequency of use (1–3, 4–7 days/week) of seven disinfectants and sprays in eight different jobs. Thus, a person with “low” exposure may be someone who uses the product infrequently whereas an individual in the “high” exposure category may be using the same product on a regular basis. It is important to note that classifications of “low”, “medium” and “high” exposures may not correspond to the actual concentrations as no measurements of chemicals were performed. Thus, while this approach provides information on frequency and duration of exposure, it does not provide information on exposure levels.

More recently, Quinot et al. [77] investigated the feasibility of using smartphones and product bar codes to advance occupational exposure assessments for cleaning and disinfecting products among hospital/cleaning workers. The smart phone application has a bar code reader and seven fields for manual data entry: product name, frequency of use (per week and per day), what product is used for, physical form of product (e.g., spray), use of protective equipment and if the product is used in a confined place. This innovative tool has the potential to advance exposure assessment but still does not provide information on concentrations of quats.

Bello et al. also used a task-based assessment method to qualitatively assess exposure to cleaning product chemicals [41]. They conducted interviews at several hospitals in eastern Massachusetts to identify the cleaning tasks and the products used. They further obtained information on products from Materials Safety Data Sheets for both concentrated and ready-to-use products, and videotaped cleaning tasks. This information was used to conduct a qualitative assessment of inhalation and dermal exposures. As in other studies described here, no environmental measurements were obtained.

A similar approach to identifying and characterizing cleaning/disinfecting tasks and products used in hospitals was described by Saito et al. [76] who monitored workers from 14 occupations at five hospitals over 216 shifts. Work tasks and products used were recorded at 5-min intervals and major chemicals in each product were identified from safety data sheets; again, no actual environmental concentration data were obtained.

The mechanisms by which quats may trigger bronchoconstriction is another area of uncertainty. It has been suggested that quats may act as pure sensitizers based on positive SIC tests [32]. However, as with many other low molecular weight compounds, the evidence of sensitization to quats is usually not expected to be supported by IgE test results [39], and as reported in the most recent update of the UK occupational surveillance data [78], not all asthma cases attributed to quats are accompanied by a positive SIC test.

Although beyond the scope of the current review, a useful insight into the relation between quats and asthma can be found in clinical studies. One specific quats compound, BAC, is routinely included in bronchodilator nebulizer solutions to prevent bacterial growth, especially in multi-dose formulations [79]. Clinical studies have shown that BAC added to nebulizer formulations may induce bronchospasms following inhalation of 300–1800 μg and the effect appears to depend on the cumulative dose [80, 81]. The mechanism of action in this case is thought to be neither sensitization nor irritation, but rather temporal increase of nonspecific bronchial reactivity (similar to but less potent than that of histamine), which increases the likelihood of bronchoconstriction in a susceptible individual [82].

Cases of paradoxical bronchospasm during nebulization treatment for asthma have been reported and attributed to BAC [83, 84], prompting calls for its removal from the available formulations [85, 86]. On the other hand, paradoxical bronchospasm has been documented even in response to bronchodilators themselves [87–89] as well as other compounds added to nebulization solution [90, 91], and even biologically inactive substances such as saline and distilled water [92, 93], underscoring the limited value of anecdotal evidence especially with respect to common multifactorial conditions such as asthma. Moreover, it appears that the likelihood of bronchospasm in response to BAC and other nebulizer additives may be dependent on the bronchodilator used for treatment [94], and bronchospasm is not observed with the use of new generation inhalation devices that tend to deliver much smaller doses of BAC and other additives [79, 95].

## Conclusion

The literature on the association between exposure to quats and asthma (either new onset or exacerbation) is limited, but has been used as the basis for the categorization of quats as occupational asthmagens. In this review, we assessed these studies by asking five key questions, and based on our assessment we draw the following conclusions:

*Does the available literature contain exposure data on specific quats? “*Quats” are not a single entity but rather a broad group of chemicals with compositions that have evolved over time. Very few studies provided information on specific compounds but rather used the broad descriptor “quats” or “BAC”. The exceptions included some case studies that contained information on the specific quat compound(s) to which individuals were exposed (lauryl dimethyl benzyl ammonium chloride [35], didecyl dimethyl ammonium chloride [33, 48, 51], dimethyl ethyl benzyl ammonium chloride [33]). However, no air concentration data were available for these studies. Thus, a critical limitation with the available studies is a lack of quantitative measures of exposure to different specific quats.

*Did the studies distinguish between exposure to quats and other agents that may cause asthma?* Cleaning/disinfecting products contain mixtures of allergens and irritants [69–71] and the activity of cleaning/disinfecting may increase intake of particles (e.g., dusts, pollen, dander, and molds) and at the same time decrease overall allergen levels [70]. While some of the studies reviewed here described various co-exposures, no reliable data pertaining to the independent effects of these co-exposures on asthma incidence are available.

*Is there a distinction between new incident asthma and exacerbation of existing condition?* The current literature often does not provide clear information about the type asthma under investigation. Even in case reports, the full asthma history is usually not available, and thus it is not clear if the majority of cases represent a new onset disease or an exacerbation of pre-existing condition. None of the population-based studies included in the current review were specifically designed to examine incident rather than prevalent asthma.

*Is there evidence that the mechanism of asthma is mediated* via *respiratory sensitivity or irritation?* The case reports and challenge studies indicate that quats may induce asthma in some individuals, as confirmed by SIC tests; however, it is still not clear if the mechanism is attributable to true sensitization or irritation with delayed onset, which may be clinically indistinguishable [32]. It is also the case that not all asthma cases attributable to BAC are confirmed by a positive SIC test. The relative frequencies of sensitization and irritation-induced asthma are important as actions taken to mitigate potential problems may differ.

*Is there evidence of a consistent association between quats and occupational asthma risk and if so, how much (or what proportion) of this risk is attributable to quats in the workplace?* While quats have been shown to induce asthma in some cases, without reliable data on the size of the exposed population from which the cases arose, case reports alone do not offer any insight into the frequency and determinants of quats-induced asthma, especially considering the widespread use of these compounds. The available population-level studies have not been properly designed to address this question and thus the contribution of quats to the overall occupational asthma burden is unknown.

In summary, the current state-of-the-science does not allow a proper assessment of the potential link between quats and occupational asthma. The unresolved methodological issues include: poor understanding of exposure pathways including the consideration that quats are non-volatile, lack of quantitative exposure data that would allow identification of an asthmagenicity threshold, insufficient information on whether quats are sensitizers or act via dose-dependent irritation or some other mechanism, and inability to quantify risk of new-onset asthma attributable to quats. Another important area of uncertainty is the lack of information on the specific quat compounds being used which effects both the exposure and toxicity potential. Finally, there is a lack of data capable of distinguishing the effects of quats from those of other chemical and biological workplace exposures. Addressing these research questions will require studies that may be both resource- and time-intensive. Yet until such studies are conducted, the existing gaps in knowledge will prevent evidence-based judgment about the comparative risks of quats and alternative methods of cleaning and disinfection.

## Data Availability

Not applicable.
